# Endodontic Flare-Ups: An Update

**DOI:** 10.7759/cureus.41438

**Published:** 2023-07-06

**Authors:** Anjali Sharma, Rohit Sharma, Madhurima Sharma, Saloni Jain, Aparna Rai, Sheersh Gupta

**Affiliations:** 1 Conservative Dentistry and Endodontics, Teerthanker Mahaveer Dental College and Research Centre, Moradabad, IND; 2 Prosthodontics and Crown and Bridge, Teerthanker Mahaveer Dental College and Research Centre, Moradabad, IND

**Keywords:** flare-up, multi-visit, single-visit, root canal treatment, pain, acute exacerbations

## Abstract

Root canal treatment deals with mechanical and chemical cleaning followed by obturation that promotes healing and repair of periradicular tissues. Flare-ups can occur in between or some days after endodontic therapy leading to unscheduled visit by the patient. This complication is characterized by severe pain and/ or swelling. There is a correlation between number of appointments, intracanal medicament used and flare-ups. However, there is no sure procedure that can avoid this complication. Therefore, this review article has discussed about causes and some procedures to prevent and treat flare-ups.

## Introduction and background

Endodontic infection is related to biofilms. The main objective of endodontic treatment is the removal of biofilms for a successful outcome. Mostly, endodontic diseases are caused by surface-related microbial growth. It is crucial to know about biofilms to understand the pathogenicity of endodontic microorganisms as well as for proper root canal disinfection. It is important to know how a biofilm that is formed by endodontic bacteria resists root canal treatment (RCT). The main cause of primary and secondary root canal infection may be attributed to biofilms of Enterococcus faecalis. Despite recent advances in endodontic instruments, instrumentation techniques, and irrigants, we are not able to completely remove endodontic biofilms due to complex root canal morphology. We are not able to reach certain areas like isthmus and fins in the root canal system which leads to incomplete removal of biofilms and increases chances of post-endodontic flare-ups. Microbes can penetrate within the root canal system before, in between, or after endodontic treatment. During treatment, microbes can enter through calculus, dental biofilms, leakage in rubber dams, caries on teeth, contamination of intracanal medicament, irrigant contamination, or contamination of endodontic instruments. Microbes can enter the root canal in between appointments by leakage or loss of temporary restoration, tooth fracture, and open dressings for drainage. Microbes can even enter after RCT by use of infected gutta-percha, loss or leakage through permanent or temporary restorative materials, delay in permanent restoration, and recurrent caries. Flare-ups are defined as the occurrence of severe pain and swelling following RCT resulting in unscheduled appointments by the patient. Patients may experience flare-ups in between or after RCT. The factors responsible for flare-ups are microbial, chemical, and mechanical injuries to the pulp. Also, there is a correlation between flare-ups and gender, age, preoperative pain, tooth type, number of visits, irrigation techniques, and use of intracanal medicament. Also, some medicines were found important to prevent flare-ups. However, there is no definite treatment protocol to prevent flare-ups. Swelling and pain (flare-ups) are usually encountered due to improper instrumentation and irrigation. Improper instrumentation and dry filing may lead to pushing of debris into periapical tissues leading to flare-ups [1].

Improper instrumentation, improper obturation, and irrigation can lead to persistence of inflammation and infection. This can further lead to periapical abscess and sinus formation. The success of therapy depends on the presence or absence of periapical lesions, vitality of the tooth, and tooth type (posterior or anterior).

The main debate in endodontic treatment is whether to go for multiple sessions or a single session. Periradicular tissue destruction and inflammation due to the presence of bacteria result in apical periodontitis. There is a fight between the immune response and microbes at the junction of pulpal tissue and periodontal ligament. This causes resorption of hard tissue and further periapical destruction leading to development of periapical lesions [1].

Single-session RCT is cost-effective, causes less anxiety of anesthesia to the patient, and is less time-consuming. The main disadvantage of single-sitting RCT is that we cannot check the tissue response before obturation. This article discusses the causes, prevention, and treatment of flare-ups [1].

## Review

Treatment protocols for multi- and single-session RCT

Periapical lesions are formed due to the persistence of bacteria within the root canal system [2]. In multiple-sitting RCT, mostly non-hard setting calcium hydroxide (Ca(OH)_2_) paste and Metapex (calcium hydroxide with iodoform) are used as intracanal medicaments in cases of periapical lesions [3].

Ca(OH)_2_ dissociates into hydroxide and calcium ions. The OH- cuts the bacterial endotoxins and chain of protein into pieces by breaking the chemical bonds. Ca(OH)_2 _breaks the protein into harmless fragments. It also dissolves the bacterial endotoxins and kills the bacteria [3].

Entombing theory is the basis of single-visit RCT. Most of the bacteria are killed while irrigation and the remaining are entombed within the root canal depriving the bacteria of nutrition thereby killing or inactivating them [4].

Challenges faced during RCT

The problems encountered during RCT include the presence of biofilms within the root canal system, complex root canal morphology, and development of a smear layer during instrumentation. Sodium hypochlorite with EDTA (ethylenediaminetetraacetic acid) has always remained the gold standard from historical times till date because of its synergistic properties to dissolve necrotic tissues, remove microorganisms, and remove the biofilm and the smear layer. 

Causes of flare-ups

Periradicular damage during RCT can lead to flare-ups. As this happens, there is development of an immune response by the body leading to pain and swelling. There are many factors that can cause flare-ups.

Mechanical factors

Mechanical factors can lead to pushing of debris and microorganisms into the periradicular space leading to pain and swelling (flare-ups). The chance of apical pushing of debris is less with the crown-down technique [5]. There are fewer chances of flare-ups with rotary instruments than manual hand K files [6]. There is a lower incidence of pain with reciprocating instruments in cases of endodontic re-treatment [7].

Wrong working length determination can also result in flare-ups [8]. Various methods are available to determine the correct working length. Overextended working length estimation can result in unwanted periapical instrumentation and periapical pushing of debris resulting in flare-ups [9]. So, correct working length determination is necessary to prevent flare-ups [10].

Patency should always be maintained for better apical cleaning, irrigation, and working of intracanal medicament, but this technique can also lead to periapical inflammation by apical pushing of debris by K files. Apical enlargement can also lead to periapical pushing of debris and development of flare-ups [11].

Microbial factors

The aim of RCT is to remove all the microorganisms from the root canal system. There is a balance between root canal microflora and the host immune system which is known as local adaptive syndrome. When there is presence of asymptomatic apical periodontitis and we are accidentally pushing debris into the root canal space, there is occurrence of flare-ups. This occurs due to disturbance in this balance [5,9].

Porphyromonas gingivalis, Porphyromonas endodontalis, Fusobacterium nucleatum, and Prevotella are the bacteria mainly associated with flare-ups in symptomatic apical periodontitis [12].

Chemical factors

If intracanal medicaments, irrigants, and sealers reach the periapical tissues, they can also cause irritation there, resulting in flare-ups [13]. The use of resin of resorcinol-formaldehyde during obturation is also associated with flare-ups in re-treatment cases [14]. According to some studies, there is no influence of the type of irrigant used for irrigation [15], whereas in some studies, it was found that there is the role of type of irrigants in flare-ups [16]. Incidences of flare-ups are lower with 5.25% NaOCl compared to 2.5% NaOCl [16].

Risk factors for flare-ups

There are two groups of risk factors associated with flare-ups: factors related to patients like general health, tooth type treated, demographics, clinical symptoms, pulpal condition, apical periodontal status and therapeutic procedures like retreatment, intracanal medicaments, and number of visits [17].

Pulpal and apical periodontium status

There is a controversy about whether there is a role of pulpal status in the occurrence of flare-ups [18-20]. Some studies say that microorganisms should be present to cause periapical inflammation. So, the rate of flare-ups should be more for non-vital teeth [5,17]. Some studies say that the rate of flare-ups is higher with vital teeth [19].

Demographics

There is a great controversy about whether there is a role of age and gender in the occurrence of flare-ups. Some say its frequency is higher at a young age [21], whereas others say an older age [22]. Some say women are at higher risk of flare-ups [21] while others say there is no role of gender [17]. Flare-ups are higher in patients with diabetes compared to non-diabetics [23].

Presence of pre-operative pain

There is strong correlation between pre-operative pain and flare-ups [24]. If microorganisms from infected root canal are forced into the periapical tissues, it can lead to the occurrence of flare-ups [5]. Also, there is a strong relationship between pre-operative fear and pain [25].

Number of appointments

Re-treatment with no clinical symptoms and treatment of vital pulp can be done in a single visit with minimal chances of flare-up [26]. Single-sitting RCT is now all possible due to digital radiography, apex locators, reciprocating instruments, and better sealers [27].

Tooth type

The cortical plate of mandibular molars is thicker. So, there are higher chances of flare-ups associated with mandibular molars [18]. It was also found in some studies that there is no correlation between the tooth type and the incidences of flare-ups [15].

Irrigation

Disinfection of root canals is achieved with the help of irrigants [28]. Apical extrusion of irrigants can result in severe pain [9]. It was also found in some studies that there is no role of the depth of insertion of the needle and occurrence of pain [29]. Mechanical activation of irrigants like EndoActivator is also useful in reducing incidences of post-operative flare-ups [30]. Also, continuous ultrasonic irrigation is found to be more effective in reducing incidences of flare-ups [31]. The use of low-level laser therapy also reduces the risk of flare-ups [32]. The use of cryotherapy also reduces the incidences of flare-ups [33]. The rate of flare-ups was most dependent on the presence of periapical lesions and pre-operative pain followed by the number of visits [34].

Intracanal medicaments

Chances of flare-ups are less with good antimicrobial intracanal medicaments [35,36]. Ca(OH)_2_ was found to have less effect to control the incidence of flare-ups. There are fewer chances of flare-ups with triple antibiotic paste (ciprofloxacin 500 mg, metronidazole 400 mg, and minocycline 100 mg) compared to Ca(OH)_2_ as the intracanal medicament [37].

Pre-operative medicine to control flare-ups

Pre-operative medicine like dexamethasone and piroxicam can prevent the occurrence of flare-ups [38]. Prednisolone can also reduce symptomatic irreversible pulpitis pain [39]. In cases of symptomatic apical periodontitis, pre-operative acupuncture (acupuncture means puncturing with a needle) is found to be effective in reducing the pain of flare-ups [40].

Post-operative medicine to reduce incidences of flare-ups

Corticosteroids are found to reduce incidences of flare-ups [41]. According to a few studies, post-operative oral methylprednisolone administration has no influence on incidences of flare-ups [42]. Post-operative photobiomodulation (PBMT) treatment can reduce incidences of post-operative flare-ups. PBMT uses sources of light like LEDs, lasers, and broadband light to reduce pain. We can use a diode laser (DL) for PBMT and bactericidal effect in endodontic treatment that accelerates the repair of the periradicular tissues. The DL at 940 nm is found very effective for biomodulation and disinfection of non-vital teeth having large periapical lesions [43].

Prevention of flare-ups

Flare-ups are multifactorial. Therefore, we have to consider preventive strategies for flare-ups. There are certain strategies to reduce flare-ups. Asepsis is the vital need of RCT to avoid flare-ups. The use of rotary-driven NiTi instruments with the crown-down technique and proper irrigation can reduce incidences of flare-ups [5]. Some devices we can use for better delivery of irrigants. The use of intracanal medicaments in between appointments and single-visit treatment has the chance of reducing flare-ups [35].

Treatment of flare-ups

Cortical Trephination

Exudates accumulated in the periradicular tissues are evacuated by creating a puncture in the cortical plate known as cortical trephination [44]. Cortical trephination is usually performed in cases of acute alveolar abscess or occurrence of pain and swelling after obturation.

Re-instrumentation

If a flare-up occurred in between the appointments, the access opening should be re-opened. Working length should be re-evaluated; patency should be checked followed by copious irrigation to remove the toxic products and microorganisms. Drainage of exudates will ultimately decrease the pressure [44].

Intracanal Medicaments

Studies have shown that flare-ups cannot be relieved or prevented by intracanal medicaments like Ledermix, iodine potassium iodide, calcium hydroxide, camphorated paramonochlorophenol, formocresol, and eugenol [45]. However, they can be prevented or reduced by using non-steroidal anti-inflammatory drugs (NSAIDs) and steroids intracanal medicaments [17]. 

Incision and Drainage (I and D)

I and D cause the release of periradicular pressure and give effective pain relief. If obturation is adequate and a flare-up has occurred after completion of RCT, incision is given on the most fluctuant part followed by establishment of drainage [45].

Drugs

Non-steroidal Anti-inflammatory Drugs

NSAIDs are widely used to treat pain of flare-ups. They are very effective to treat pain of periradicular and pulpal origin [46]. Pre-operative NSAIDs can significantly reduce Prostaglandin E2 (PGE2) in patients of symptomatic irreversible pulpitis, thereby reducing the chances of flare-ups. If flare-up pain is not controlled by using NSAIDs, narcotic analgesics are prescribed alone or in combination with NSAIDs [47].

Antibiotics

Antibiotics should only be prescribed if the need arises. Unwanted prescriptions of antibiotics can lead to development of antibiotic-resistant bacteria [48]. Antibiotics should only be prescribed in cases of systemic manifestations like lymphadenopathy and fever.

Occlusal reduction

The literature does not suggest occlusal reduction for post-endodontic pain control. According to a few studies, inflammatory mediators lead to stimulation of periradicular nociceptors. Therefore, occlusal reduction can decrease the mechanical stimulation and thereby incidences of flare-ups [49].

Algorithm for management of flare-ups

When the patient experiences severe pain or swelling following or in between RCT, it is known as a flare-up which requires immediate management and results in unscheduled visits by the patient. Treatment of flare-ups includes establishment of drainage, occlusal relief, intracanal medicament, and the use of systemic medication.

## Conclusions

Flare-ups are multifactorial in origin and can occur due to chemical, mechanical, and microbial factors. Since they are multifactorial, we cannot guarantee a particular treatment strategy to prevent flare-ups. Further studies are required to develop a procedure to prevent flare-ups.
